# Analysis and Validation of Lightweight Carriage Structures Using Basalt Fiber Composites

**DOI:** 10.3390/ma17235723

**Published:** 2024-11-22

**Authors:** Xianglin Wang, Shaoqing Yuan, Wei Sun, Wenfeng Hao, Xufeng Zhang, Zhongjia Yang

**Affiliations:** 1 Sichuan Basalt Fiber New Material Research Institute, Guang’an 638500, China; 2 College of Mechanical Engineering, Yangzhou University, Yangzhou 225000, Chinahaowenfeng2007@163.com (W.H.); 3 College of Materials, Beijing Institute of Technology, Beijing 100081, China; 4 College of Chemistry, Beihang University, Beijing 100191, China

**Keywords:** composite material carriage, lightweight, finite element simulation, reliability validation analysis

## Abstract

With the growth in road transport volume and increasingly stringent environmental regulations, the use of lightweight dump trucks not only reduces fuel consumption but also enhances transport efficiency, aligning with the principles of green development. It has now become a key focus in the field of heavy-duty vehicle research. The carriage is located at the rear of the dump truck, connected to the chassis, and serves as the box for carrying cargo, making its strength and durability crucial. As one of the important components of heavy-duty vehicles, the carriage accounts for 15% to 25% of the total vehicle weight, and its weight reduction efficiency is significantly higher than that of other vehicle systems. This paper presents a prefabricated carriage structure based on basalt fiber composite panels combined with a metal frame, achieving the lightweight design of the carriage while meeting the stringent requirements for high load-bearing capacity and strength in heavy-duty vehicles, and significantly improving assembly and production efficiency. Given the complex working environment and diverse loading demands of heavy vehicles, this study incorporates real operating conditions of dump trucks, utilizing theoretical calculations and design analyses to construct finite element models for various scenarios, followed by detailed numerical simulations in ABAQUS (2023). Additionally, a bending–shear test of the side panel was designed and conducted to validate the accuracy of the finite element model, with comparative analysis performed between simulation results and experimental data, effectively assessing the safety and reliability of this lightweight composite carriage structure. The results indicate that the designed carriage not only meets the strength, stiffness, and impact resistance requirements of current heavy-duty carriages but also significantly reduces the carriage weight. This research provides scientific reference and engineering value for the application of composite materials in the lightweight design and structural optimization of dump trucks.

## 1. Introduction

With the rapid development of the domestic economy, national investment in urban and large-scale infrastructure has been increasing annually, driving the demand for load-shifting vehicles and creating significant market opportunities. Research indicates that reducing a laden vehicle’s weight by 10% can increase its range by 5–8%, improve acceleration by 8%, reduce energy consumption by 8%, and shorten braking distance by 5% [1]. Traditional freight structures are typically designed with a focus on high strength, primarily using metal materials like steel, emphasizing load-bearing capacity and structural rigidity, but they are relatively heavy. In contrast, current lightweight carriages utilize advanced composite materials and topology optimization, balancing strength and weight to achieve energy efficiency optimization [2,3,4]. Currently, the compartment structures of heavy-duty vehicles are primarily composed of welded thick steel plates, accounting for about 30% of the vehicle’s total weight, making compartment lightweighting a highly effective approach for overall weight reduction [5]. Joel et al. [6] explored weight reduction in truck trailers through material selection and structural optimization, designing a parametric finite element model for a steel trailer that demonstrated potential for achieving lightweight designs. Wang et al. [7] proposed a hybrid method based on improved NSGA-II and TOPSIS methods for optimizing a subframe lightweight design, focusing on multi-objective optimization to meet strength and stiffness requirements while reducing weight. Zaripov et al. [8] investigated the feasibility of using polymer materials for freight wagon compartments, proposing a method for manufacturing carriages with fiberglass composites and detailing relevant molding and reinforcement techniques. Peter et al. [5] achieved weight reduction by altering the cross-sectional shape and size of a truck frame, using a simplified frame model to estimate potential weight savings and applying topology optimization for geometric improvements. Sun et al. [9] employed a fuzzy multi-objective particle swarm algorithm to optimize a frame structure, successfully reducing weight while significantly improving bending stiffness, overall strength, fuel efficiency, and safety.

Replacing steel plates in vehicle compartments with lightweight materials, combined with effective structural design, is a viable solution for achieving weight reduction [10,11]. Basalt fiber-reinforced composite laminates offer high strength, stiffness, and low weight, and basalt fiber—a new, high-strength, low-cost, and environmentally friendly material [12]—aligns with global sustainability goals. This makes it a promising material for lightweight compartment design in load-shifting vehicles [13].

**Figure 1:**
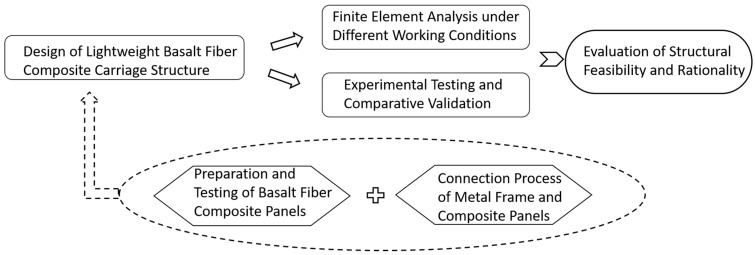
Research content flowchart.

As shown in Figure 1, this paper proposes a novel prefabricated dump truck carriage structure based on basalt fiber composites. This design not only effectively achieves the goal of a lightweight carriage but also meets the stringent performance requirements for load-bearing capacity and strength in heavy-duty carriages. To validate the actual working performance of the lightweight basalt composite carriage, various operating conditions were established, and a systematic analysis of the overall structure was conducted through theoretical calculations, simulation analysis, and experimental verification. A comparison with traditional metal carriages further confirms the rationality and feasibility of this design [14]. This study aims to analyze and validate the feasibility of using basalt fiber composites in carriage applications, providing important theoretical foundations and practical guidance for the lightweight design and structural optimization of dump trucks.

## 2. Design, Simulation, and Experimental Methods

### 2.1. Structural Design

**Figure 2:**
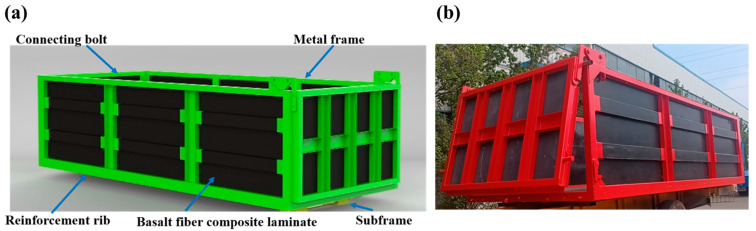
(**a**) 3D model of basalt fiber composite dump truck carriage; (**b**) Production image of dump truck carriage.

As shown in Figure 2a, the designed lightweight carriage consists primarily of a metal frame and basalt fiber composite laminates. The designed carriage has an internal length of L = 5800 mm, internal width of W = 2300 mm, and an internal height of H = 1500 mm. The metal frame is made of T700 steel (Shanxi Taigang Stainless Steel Co., Ltd., Taiyuan, China). The basalt fiber composite laminate employs a multilayer hybrid structure, where resin (Beijing Institute of Technology, Beijing, China) is evenly applied to the fiber fabric to ensure the complete wetting of each fiber. The basalt fiber plain weave fabric, unidirectional fabric, and multiaxial fabric (+45°/−45°) (Sichuan Qianyi Composites Co., Ltd., Huaying, China) are laid up sequentially. The impregnated composite materials are placed in a mold, utilizing a hot press curing process with a temperature gradient of 80 °C for 30 min, followed by 90 °C for 10 min, and finally held at 130 °C for 120 min, with a pressure set at 0.4 MPa and a heating rate of 1.2 °C/min. To further mitigate the impact of cargo loading on the basalt fiber composite panels, a protective layer made of high-density polyethylene is applied to the outer wall of the laminate to disperse the impact load from the cargo, preventing damage to the laminate due to excessive impact.

Given the unique properties of fiber-reinforced composites, traditional metal joining methods such as welding or drilling cannot be utilized. Therefore, a pin connection structure combined with the metal frame is employed in the design process. Specifically, the carriage panels are segmented into multiple modules that are embedded within the metal frame. High-strength bolts of grade 8.8 are selected to fasten the metal frame, achieving clamping of the composite laminate panels. Subsequently, adhesive is applied to secure the connection, ensuring the stability of the panel–frame interface.

This design achieves significant weight reduction while meeting the high load-bearing and strength requirements of heavy-duty compartments [15]. The modular approach not only enhances assembly and production efficiency but also improves the maintainability of the carriage, reduces maintenance costs, and allows for the replacement of damaged panels individually. Figure 2b shows the overall display of the carriage after assembly production.

### 2.2. Material Properties

**Figure 3:**
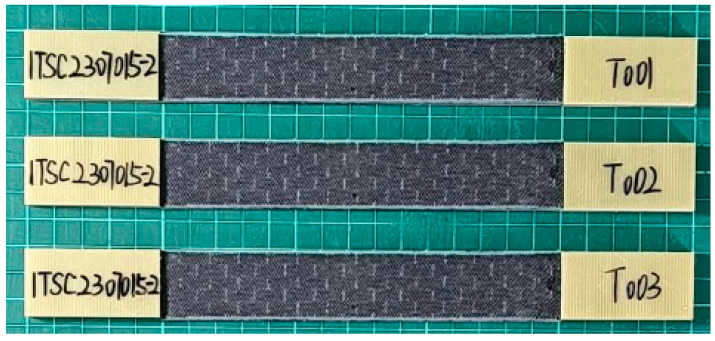
Three standard tensile specimens.

**Figure 4:**
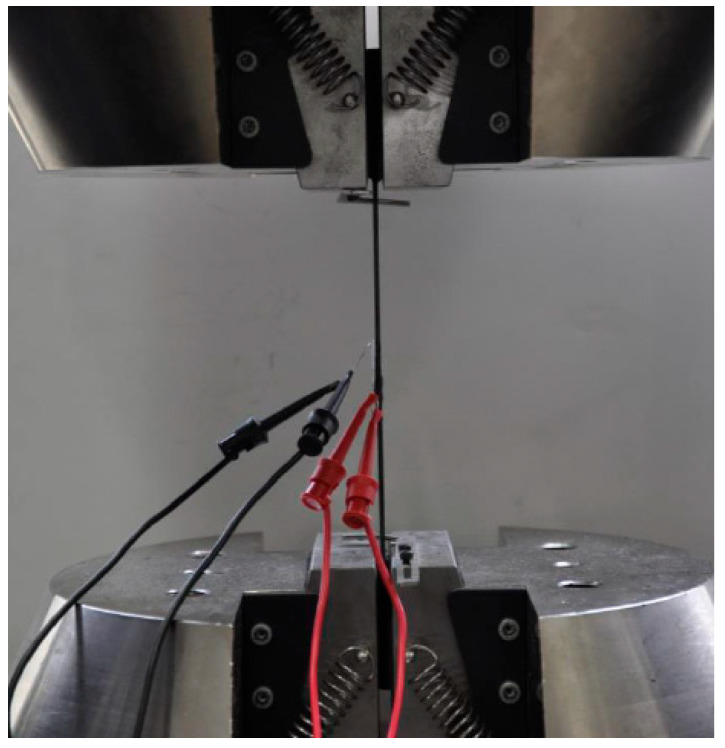
Standard specimen tensile test.

According to the ISO 527-4 standard [16], basalt fiber composite boards were fabricated into standard test specimens, as illustrated in Figure 3, and their material properties were determined through tensile testing [17]. As shown in Figure 4, the tests were conducted using an electronic universal testing machine (INSTRON 5985, Boston, MA, USA.) at a tensile speed of 2 mm/min, with specimen dimensions of 250 × 25 mm, Strain gauges (BE120-2AA, Zhonghang Electronic Measuring Instruments Co., Ltd., Hanzhong, China) are used to record strain in real-time. To ensure data accuracy, three separate tests were performed, and the resulting material parameters are summarized in Table 1. The mechanical property data of the basalt fiber composite laminate and the T700 high-strength steel used for the metal frame obtained from the experiments are presented in Table 2.

### 2.3. Working Condition Settings

Based on the actual working conditions of the load compartment, its typical operational scenarios can be categorized into three main conditions: static, dynamic (which includes full-load start acceleration, full-load braking, full-load direction changes during cornering, and full-load cornering braking), and cargo impact. In the following sections, the loads for finite element simulations under these three typical conditions are calculated. The physico-mechanical properties of the 40-ton sandy soil cargo are assumed to be as follows: soil density ρ=2.6 g/cm³, internal friction angle φ=40°, and Rankine earth pressure coefficient Ka=tan²(45°−φ / 2).

Total volume of sand and soil:

(1) $$V_{total}=\frac{m}{\rho }\approx 7.692\;m^{3}$$

Carriage floor surface area:

(2) $$A_{1}=L\times W=5.8\times 2.3=13.34\;m^{2}$$

The height of the ore:

(3) $$h=\frac{V_{total}}{A_{1}}=\frac{16.67}{13.34}=1.25\;m$$

#### 2.3.1. Working Condition 1: Static Condition

Owing to the complex mechanical characteristics of the loaded cargo, the force exerted on the bottom plate is simplified as hydrostatic pressure [18], while the pressures on the side, front, and rear plates are determined based on the Rankine earth pressure theory:

The pressure of the cargo on the base plate:

(4) $$P_{1}=\rho gh\approx 0.029$$

The pressure of the cargo on the side panels:

(5) $$P_{2}=P_{1}\times K_{a}=\rho gh\times {tan}^{2}\left(45{}^{\circ}-\frac{\varphi }{2}\right)=0.0142\;MPa$$

#### 2.3.2. Working Condition 2: Dynamic Condition

(1) Full-load start-up acceleration condition:

Acceleration $a_{1}=3\;m/s^{2}$ replaces the action of the carriages and cargo due to the inertial forces of start-up acceleration;

(2) Full-load brake operating conditions:

Braking acceleration $a_{4}=5\;m/s^{2}$ occurs instead of inertial forces of the carriages and cargo due to start-up acceleration; vertical acceleration $a_{5}=1.2\times 9.8=11.76\;m/s^{2}$;

(3) Full-load turning condition:

A lateral acceleration $a_{2}=0.3\times 9.8=2.94\;m/s^{2}$ instead of the effect of inertial force of the carriages and cargo due to turning direction; vertical acceleration $a_{3}=2\times 9.8=19.6\;m/s^{2}$;

(4) Full-load cornering brake condition:

The loaded vehicle may be subjected to sharp braking and steering in real road traffic, so the working condition is used to simulate the limiting use of the compartment [19]. According to the balance of forces and moments, the acceleration in three directions can be calculated, taking the lateral acceleration as $a_{21}=2.94\;m/s^{2}$, taking the braking acceleration in the traveling direction as $a_{4}=5\;m/s^{2}$, and taking the vertical acceleration as $a_{3}=19.6\;m/s^{2}$.

#### 2.3.3. Working Condition 3: Impact Condition

Assuming the weight of the ore ranges between 0.05 and 0.2 tons, it is generally considered that the carriage is initially empty, meaning that when the ore is loaded for the first time, there is no cushioning effect, resulting in the largest impact load.

Therefore, this analysis focuses solely on the impact load from the ore, excluding the static pressure generated by the ore’s mass on the carriage. The impact load is primarily determined by the velocity of the ore when it strikes the carriage [20], which can be calculated based on the velocity generated by free-fall motion:

(6) $$v=\sqrt{2gh_{max}}\;\approx \;6.42\;m/s$$

where g is the acceleration of gravity taken as $9.8\;m/s^{2}$ ; $h_{max}$ is the maximum height of the side of the wagon plus 500 mm.

### 2.4. FE Analysis

**Figure 5:**
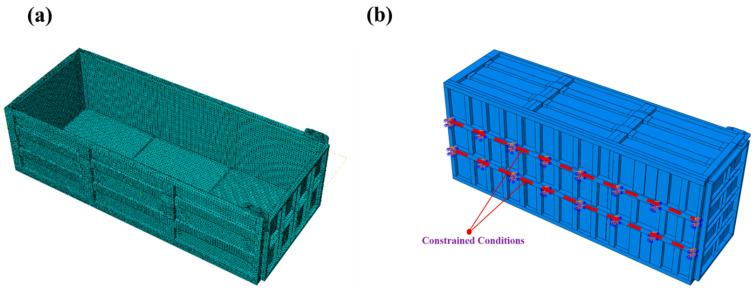
(**a**) Finite element model mesh diagram of the carriage; (**b**) Boundary condition settings.

To facilitate meshing and finite element analysis in ABAQUS (2023), the compartment is simplified to consist of basalt fiber composite laminates, a metal frame, reinforcements, and bolts. In this study, the 3D model of the compartment, created in SolidWorks (2023), was saved in IGS format and subsequently imported into ABAQUS (2023), for structural simplification and meshing. As shown in Figure 5a, a schematic of the finite element model setup and meshing is presented. Due to the complexity of the carriage structure, a linear tetrahedral mesh of type C3D4 is employed, resulting in a total of 101,844 nodes and 425,725 elements.

During operation, the composite compartment is held in place by a subframe that is fixed to the load vehicle. Therefore, the subframe can be fully constrained, ensuring that it remains completely stationary, following its working conditions. Based on the various working conditions outlined in the previous section, equivalent forces are applied to the compartment model, as shown in Figure 5b. The composite laminate is inserted into the grooves of the metal frame and adhesively bonded at the seams; therefore, the contact interface between the basalt fiber composite panel and the metal frame is simplified and modeled as bonded. The bolts on the metal frame apply a preload to clamp the frame, securing the composite panel in place. Thus, a preload is applied at each bolt location. The Hashin criterion, due to its effectiveness in addressing the anisotropy and complex failure modes of composite materials, is suitable for the basalt fiber composite panels involved in this study. Therefore, the Hashin failure criterion [21] is employed to simulate damage evolution. Loads are applied according to the design specifications for each working condition. In static conditions, the theoretically calculated pressure is applied to the side walls and bottom of the carriage. In dynamic conditions, equivalent accelerations are applied to the panels of the carriage for analysis and computation. When the carriage experiences impact conditions, the cargo is modeled as a rigid body, and the corresponding velocity is applied to simulate the impact on the bottom panel. Additionally, each simulation calculation must also consider the self-weight of the carriage and the weight of the cargo.

### 2.5. Experimental Design and Validation

Due to the large overall volume of the designed compartment structure, conducting a full-scale experiment is impractical. Therefore, the side panels that exhibited the most significant deformation during the simulation were selected as representatives for the compression experiment. In this experiment, the side panels and their corresponding metal frames were fabricated to the same scale, and the results from the finite element analysis were compared with experimental outcomes. This comparison was performed to ensure the validity of the experimental design and to verify the accuracy of the finite element analysis.

**Figure 6:**
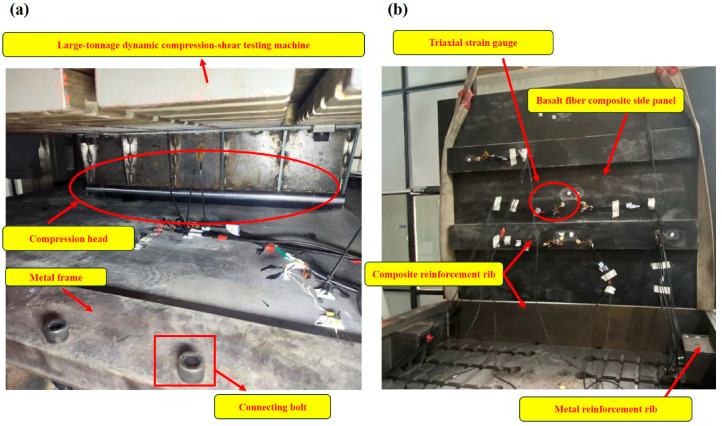
Test site diagrams; (**a**) Bending Resistance Mechanical Performance Testing of the side panel; (**b**) Strain gauge data collection diagram.

As shown in Figure 6a, the basalt fiber composite side panels, along with the metal frame, were subjected to bending resistance tests using a multifunctional static compression and shear testing machine (SYWY-2000JD, Jinan Sanyue Testing Instrument Co., Ltd., Jinan, China). The tests were conducted along the midline direction of the side panel, perpendicular to the ribs, and displacement-load test data were collected during the bending resistance performance testing, with curves plotted [22]. Downward displacement was applied from the top at a ram speed of 5 mm/min. A force value was recorded for every 5 mm of displacement, with the maximum force noted until the test piece experienced compression failure. Figure 6b illustrates the strain gauge acquisition system, utilizing the DH3816N (measurement range of strain: ±60,000 με, accuracy 0.1 με) stress strain test and analysis system to measure strain at designated points on the side panel through strain gauges [23]. These results were then compared with the simulation data for validation.

## 3. Results & Discussion

### 3.1. Finite Element Results Analysis

**Figure 7:**
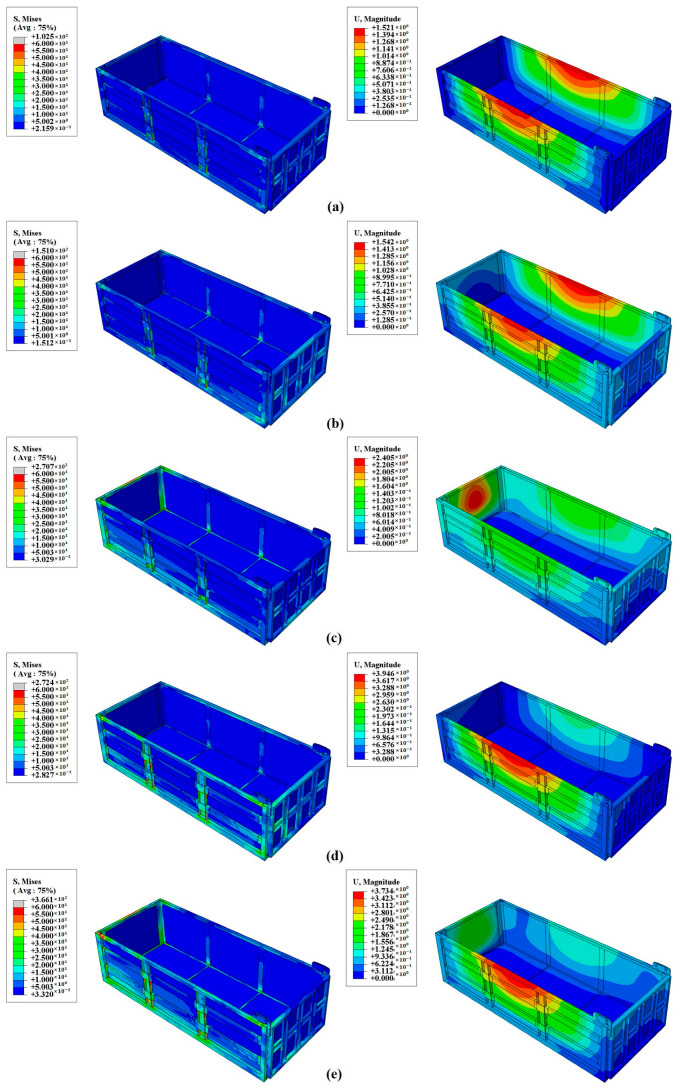
Stress–Strain nephogram of the carriage under different conditions; (**a**) Static condition; (**b**) Full-load start-up acceleration condition; (**c**) Full-load braking condition; (**d**) Full-load turning condition; (**e**) Full-load turning braking condition.

**Figure 8:**
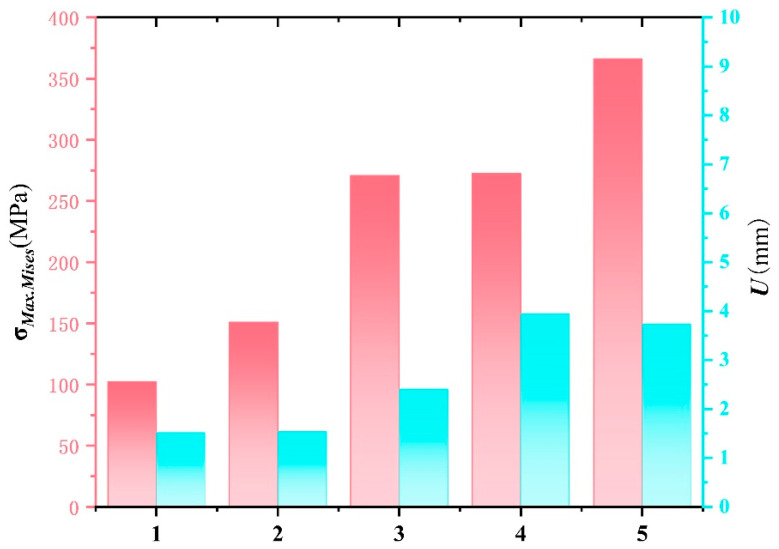
Maximum stress and strain values under different conditions. Note: (1) Static condition; (2) Full-load start-up acceleration condition; (3) Full-load braking condition; (4) Full-load turning condition; (5) Full-load turning braking condition.

Figure 7 presents the finite element stress and deformation maps of the composite compartment under five different operating conditions: static, dynamic (full-load starting acceleration, full-load braking, full-load direction change during turning, and full-load turning braking). Figure 8 summarizes the maximum stress and displacement values for each condition. From these figures, it is evident that both the loads and deformations experienced by the composite compartment increase progressively as the working conditions become more demanding.

Most of the stress is borne by the metal frame, with concentrations primarily in the two vertical beams of the side panels, the upper beams of the side panels, and the upper beams of the rear panels. In stationary and starting conditions, the compartment experiences relatively low stress and deformation. During braking, the inertia force of the cargo results in greater stress and deformation on the front panel. In turning conditions, taking a right turn as an example, centrifugal force causes the cargo to tilt left, exerting pressure on the left side panel with the squeezing force concentrated on the left side. The maximum stress recorded is 272.4 MPa, located at the vertical rib of the side panel, a part of the metal frame locally reinforced with T700 steel, which ensures it meets design requirements without risk of failure.

Under the most severe condition—full-load turn braking—the compartment experiences double compression from the cargo, primarily affecting the front and left sides. The front side panel endures the most stress and deformation. The maximum deformation is 3.73 mm, occurring at the top center of the side panel. The maximum stress, recorded at 366.1 MPa, is located at the vertical reinforcement of the side panel. This value remains approximately 300 MPa below the permissible stress of T700 steel, indicating that the structural design meets safety requirements under real-world working conditions.

**Figure 9:**
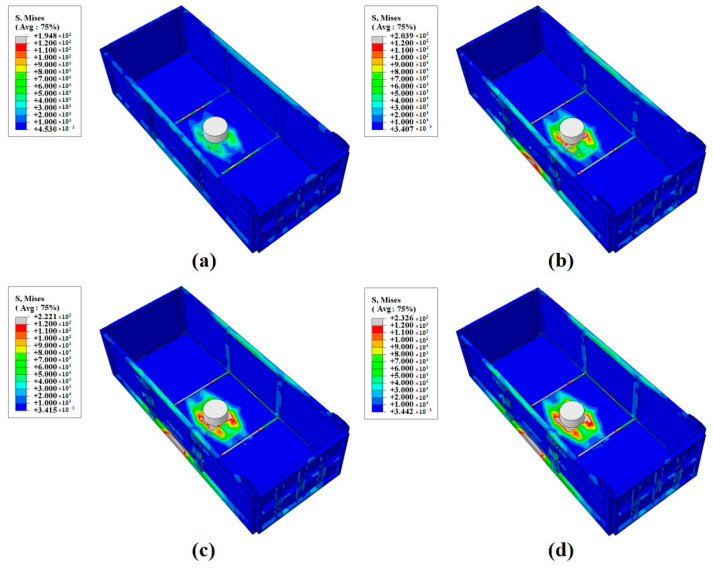
Impact of different loads:(**a**) 0.05 t; (**b**) 0.1 t; (**c**) 0.15 t; (**d**) 0.2 t.

When subjected to impact conditions, the maximum stresses in the compartment are typically concentrated at the point of impact or at the junction between the longitudinal beam and the compartment floor, which are generally the structural weak points. Figure 9 illustrates the stress–strain curves for the composite compartment under the impact of different ore weights, while Table 3 provides a summary of the maximum stress values under various loads. According to the data, as the mass of the impacting ore increases, the maximum stress on the compartment floor also rises, which aligns with real working conditions. Under the impact of a 0.2-ton ore block, the maximum stress reaches 232.6 MPa, with the stress concentrated at the metal reinforcement in the composite material of the floor plate. However, this stress level remains below the maximum allowable stress for the compartment material, indicating that the compartment structure can meet operational requirements and ensure safety and stability under these impact conditions.

### 3.2. Comparison and Validation of Experimental Results

**Figure 10:**
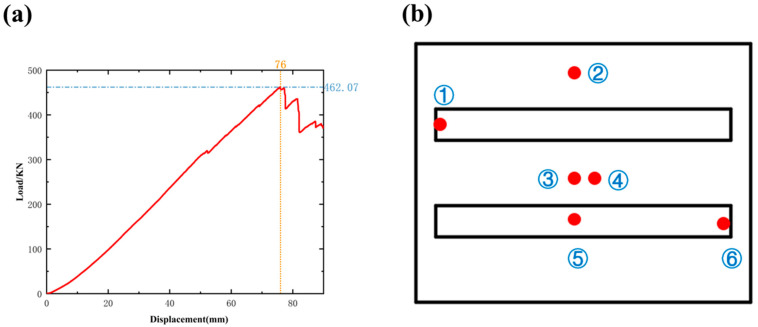
(**a**) Experimental compression load-displacement curve diagram; (**b**) Stocking film layout point.

The finite element analysis results indicate that the designed composite compartment structure operates within the permissible stress limits of the material under all typical working conditions, demonstrating that it meets the required standards for strength and stiffness in practical applications. Among the various parts of the compartment, the side panels experience the highest stresses and deformations, making them a critical focus for further experimental analysis. This additional investigation aims to assess the overall feasibility of the compartment structure and verify the accuracy of the finite element analysis results. The load-displacement curves obtained from the experiment are shown in Figure 10a. When the displacement reached 76 mm, the structure achieved its maximum load capacity of 462.07 kN. However, as the displacement continued to increase, the load-bearing capacity of the side panels decreased rapidly, indicating that the structure had sustained damage and lost its load-bearing ability [24]. These experimental results provide insight into the failure mode of the side panels under extreme working conditions and offer a foundation for future optimization of the design. During the compression tests, strain was measured in three directions using tri-directional strain gauges, and the measurement points are depicted in Figure 10b, obtaining $\epsilon_{1}$, $\epsilon_{2}$, and $\epsilon_{3}$ using the elastic modulus of the material E and Poisson’s ratio ν, while the principal stress is calculated by the strain–stress relationship:

(7) $$\sigma_{1}=\frac{E}{1-\nu^{2}}\left(\epsilon_{1}+\nu \epsilon_{2}\right)$$

(8) $$\sigma_{2}=\frac{E}{1-\nu^{2}}\left(\epsilon_{2}+\nu \epsilon_{1}\right)$$

(9) $$\sigma_{3}=\frac{E}{1-\nu^{2}}(\epsilon_{3}+\nu (\epsilon_{1}+\epsilon_{2}))$$

The calculated principal stresses are then substituted into the equivalent force equation to calculate the equivalent force:

(10) $$\sigma_{e}=\sqrt{\frac{1}{2}\left[(\sigma_{1}-\sigma_{2}{)}^{2}+(\sigma_{2}-\sigma_{3}{)}^{2}+(\sigma_{3}-\sigma_{1}{)}^{2}\right]}$$

Among them$,\;\sigma_{e}$ is the equivalent stress (von Mises stress), and $\sigma_{1}$, $\sigma_{2}$, and $\sigma_{3}$ together represent the principal stress [25].

The calculated equivalent stresses at each point are summarized in Table 3. It can be observed that the maximum stresses are concentrated at points 3 and 5, which are located directly beneath the indenter. Point 3, positioned at the center of the composite laminate, bears the highest stress of 334.09 MPa. Point 5, located on the reinforcing bar, exhibits even higher stress than point 3, indicating that the reinforcing bar is capable of withstanding greater stress loads. Point 1 is situated on the metal frame, while point 6 is at the edges of the composite sheet and metal frame reinforcement. The stress values at points 1 and 6 are comparatively lower, especially at point 1, where the higher strength of the metal frame results in relatively lower stress due to its reinforcement. Overall, the stress levels at all points remain below the allowable stress limits of the materials used, demonstrating that the structure has sufficient safety margins and is well suited for meeting practical working requirements.

**Figure 11:**
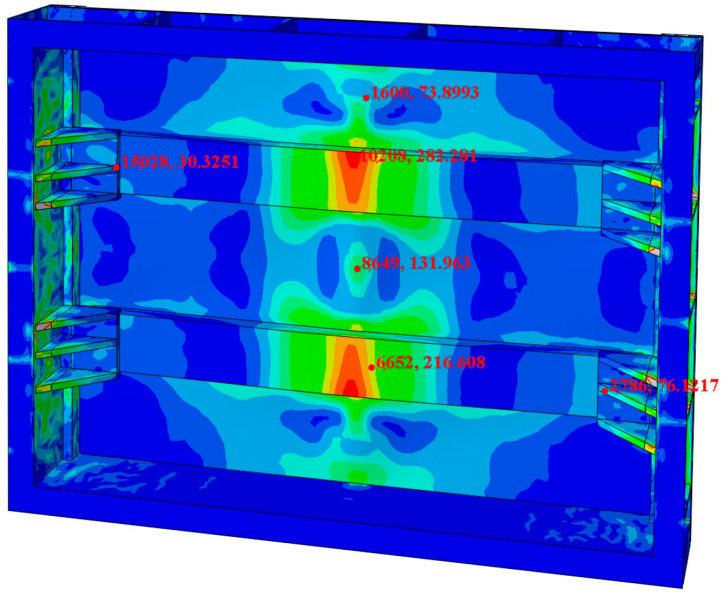
Stress values at different points in the finite element nephogram.

By comparing and analyzing the experimental data with the finite element analysis results, the validity of the compartment structure design can be further confirmed. Figure 11 illustrates the corresponding positions of the experimental collection points in the finite element stress cloud map. The finite element calculation values at these points were compared with the experimental data, as summarized in Table 4. Due to potential errors in machining accuracy and other experimental factors, the stress values from the finite element calculations were generally lower than those measured experimentally. However, the average error between the two sets of data is 11.1%, which falls within the acceptable range. This demonstrates the high accuracy of the finite element model and further validates the experimental design.

## 4. Conclusions

In this paper, the structure of basalt fiber lightweight composite compartments was designed, and various typical working conditions were established based on the actual operating conditions of dump trucks. The finite element model was systematically analyzed using ABAQUS (2023), to assess the forces acting on the structure, and the corresponding equivalent stress cloud diagrams were generated. These results were further analyzed and validated through experimental testing. The key conclusions drawn from this study are as follows:

(1) A novel prefabricated dump truck carriage structure based on basalt fiber composites is proposed, effectively achieving the goal of being lightweight while meeting the stringent performance requirements for load-bearing capacity and strength in heavy-duty carriages. Key parameters of the basalt fiber composite were obtained through experiments, and the carriage was subjected to stress analysis under six different operating conditions to systematically evaluate its feasibility.
(2) The maximum stress occurs in the reinforcing bar of the T700 metal frame under the full-load turning braking condition, with a value of 366.1 MPa. The maximum displacement of 3.73 mm occurs in the side panel during the full-load direction change condition. Both values meet design requirements, confirming the structural feasibility of the composite compartment.
(3) By conducting bending resistance tests on the side panels subjected to greater forces and comparing the results with finite element analysis, an error of 11.1% was obtained, indicating a good consistency between the two and further validating the accuracy of the finite element analysis of the overall structure. The overall structure of the carriage can be further optimized for weight reduction through methods such as topology optimization. Additionally, incorporating vehicle test data under subsequent actual working conditions will provide strong support for further improvements to the finite element model.

## Figures and Tables

**Table 1:** Material properties of the three standard parts.

NO.	*b* (mm)	*H* (mm)	$F_{m}$ (KN)	$\sigma_{m}\;(MPa)$	E (GPa)	μ
01#	25.06	2.261	57.26	1010.59	38.53	0.3
02#	25.06	2.261	60.23	1063.06	36.99	0.3
03#	25.06	2.261	57.67	1017.82	37.76	0.3

Note: b: width; H: thickness; $F_{m}$: maximum load; $\sigma_{m}$: tensile strength; E: tensile modulus; μ: poisson’s ratio.

**Table 2:** Mechanical parameters of basalt fiber composite materials and metallic materials.

	E (GPa)	$\sigma_{m}\;(MPa)$	μ
Basalt fiber composite	37.75	1039.28	0.3
T700	169	700	0.305

**Table 3:** Maximum stress values of the floor under different impact loads.

Load (t)	$\sigma_{Max.Mises}\;(MPa)$
0.05	194.8
0.1	203.9
0.15	222.1
0.2	232.6

**Table 4:** Comparison of strain gauge experimental data with finite element analysis.

NO.	1	2	3	4	5	6
Test value (MPa)	33.61	80.43	334.09	164.56	218.7	87.33
FEA (MPa)	30.33	73.90	282.28	131.96	216.61	76.12
Error rate	9.9%	8.1%	15.5%	19.8%	0.9%	12.8%

## Data Availability

The original contributions presented in this study are included in the article. Further inquiries can be directed to the corresponding author.

## References

[1] Woodrooffe J. (2016). Opportunity Cost for Society Related to U.S. Truck Size and Weight Regulation: Freight Efficiency. Transp. Res. Record.

[2] Phiri R., Mavinkere Rangappa S., Siengchin S., Oladijo O.P., Ozbakkaloglu T. (2024). Advances in Lightweight Composite Structures and Manufacturing Technologies: A Comprehensive Review. Heliyon.

[3] Lan N., Wang Q., Deutz P. (2024). Chinese transport emissions reduction policies: Analysis of purchase intention and approaches to promote uptake of new energy construction dump trucks. J. Clean. Prod..

[4] Hagnell M.K., Kumaraswamy S., Nyman T., Åkermo M. (2020). From aviation to automotive—A study on material selection and its implication on cost and weight efficient structural composite and sandwich designs. Heliyon.

[5] Seyfried P., Taiss E.J.M., Calijorne A.C., Li F.-P., Song Q.-F. (2015). Light weighting opportunities and material choice for commercial vehicle frame structures from a design point of view. Adv. Manuf..

[6] Galos J., Sutcliffe M. (2020). Material Selection and Structural Optimization for Lightweight Truck Trailer Design. SAE Int. J. Commer. Veh..

[7] Wang D., Jiang R., Wu Y. (2016). A hybrid method of modified NSGA-II and TOPSIS for lightweight design of parameterized passenger car sub-frame. J. Mech. Sci. Technol..

[8] Zaripov R., Gavrilovs P. (2017). Research Opportunities to Improve Technical and Economic Performance of Freight Car through the Introduction of Lightweight Materials in their Construction. Procedia Eng..

[9] Ren M., Sun T., Shi Y., Zheng S., Li Y. (2019). Lightweight optimization of vehicle frame structure based on the kriging approximate model. J. Mech. Strength.

[10] Mohrbacher H., Spöttl M., Paegle J. (2015). Innovative manufacturing technology enabling light weighting with steel in commercial vehicles. Adv. Manuf..

[11] Zhang W., Xu J. (2022). Advanced lightweight materials for Automobiles: A review. Mater. Des..

[12] Chowdhury I.R., Pemberton R., Summerscales J. (2022). Developments and Industrial Applications of Basalt Fibre Reinforced Composite Materials. J. Compos. Sci..

[13] Pan F., Zhu P., Zhang Y. (2010). Metamodel-based lightweight design of B-pillar with TWB structure via support vector regression. Comput. Struct..

[14] Li Y., Lin Z., Jiang A., Chen G. (2004). Experimental study of glass-fiber mat thermoplastic material impact properties and lightweight automobile body analysis. Mater. Des..

[15] Singh H., Singh Brar G., Kumar H., Aggarwal V. (2021). A review on metal matrix composite for automobile applications. Mater. Today: Proc..

[16] (2018). Plastics—Determination of Tensile Properties—Part 4: Test Conditions for Isotropic and Anisotropic Fibrous Composites.

[17] Ram K., Gupta M., Kartikeya K., Khatkar V., Mahajan P., Bhatnagar N. (2024). Performance analysis of fiber-reinforced polypropylene composite laminates under quasi-static and super-sonic shock loading conditions for impact application. J. Compos. Mater..

[18] Wan Q., Ruan J. (2020). Structural Optimization Design of a Dump Truck Cargo Compartment Based on Hybrid Sensitivity Analysis. Sci. Technol. Eng..

[19] Hu D., Chen Y. (2022). Finite Element Analysis of the Strength of Electric Wheel Dump Truck Cargo Compartment. Intern. Combust. Engine Parts.

[20] Ren X., Wang M., Gao Y. (2009). Strength Analysis of Mining Truck Cargo Compartment under Ore Impact Load. Constr. Mach..

[21] Hashin Z. (1980). Failure Criteria for Unidirectional Fiber Composites. Journal of Applied Mechanics..

[22] Hsieh T.-H., Wang I.-H., Huang Y.-S., Tsai S.-N. (2023). Repairing damaged composite laminates using carbon fiber reinforced bulk mold compound. Mod. Phys. Lett. B.

[23] Alci M., Gunes R. (2023). A comparison study on experimental characterization of unidirectional fiber reinforced composites using strain-gauges and virtual extensometers. Mater. Test..

[24] Tuo H., Lu Z., Ma X., Xing J., Zhang C. (2019). Damage and failure mechanism of thin composite laminates under low-velocity impact and compression-after-impact loading conditions. Compos. Part B Eng..

[25] Maharshi K., Patel S. (2024). Experimental and Numerical Analysis of Lightweight Hybrid Composites Under Low Velocity Impact. Appl. Compos. Mater..

